# The relationship between social identification and local voting, and its interplay with personal and group discrimination among the descendants of Turkish immigrants in Western Europe

**DOI:** 10.1186/s40878-018-0087-1

**Published:** 2018-08-16

**Authors:** Maria Kranendonk

**Affiliations:** University of Amsterdam, Nieuwe Achtergracht 166, Amsterdam, 1018 WV The Netherlands

**Keywords:** Social identification, Perception of group discrimination, Personal discrimination, Descendants of immigrants, Local voting

## Abstract

This study explores how Turkish and Islamic identifications relate to local voting likelihood among the descendants of Turkish immigrants in 10 Western European cities using *The Integration of the European Second Generation* (TIES) survey data (Herzog-Punzenberger, 40 Jahre und eine Generation später – die Kinder der angeworbenen Arbeitskräfte in Österreich sind erwachsen, 2010; Crul et al., The European Second Generation. Does the Integration Context Matter?, 2012; and Fibbi et al., The new second generation: Youth of Turkish and former Yugoslav descent in Zurich and Basel, 2015). Unlike previous studies of the politicization of social identification, it researches local voting and considers how this relationship is moderated by the interplay between perceptions of personal discrimination and group discrimination. Islamic identification relates negatively to local voting likelihood among Muslims who perceive both high levels of personal and group discrimination. This study concludes that it is crucial to take the interplay between perceived personal discrimination, perceived group discrimination, and the countries’ policy context into account in studying the politicization of social identification.

## Introduction

Immigrants and their descendants from predominantly Muslim countries are regularly confronted with an ascribed ‘Muslim identity’ and discrimination toward them personally and toward Muslims in general. The rise of anti-immigrant parties (such as the PVV— Freedom Party—in the Netherlands) is also accompanied with ‘Muslim immigrants’ being more often subject to political claims (Berkhout & Ruedin, 2017). 9/11 led to more Islamophobia in some European countries (e.g. Fisher, Heath, Sanders, & Sobolewska, 2015 for the U.K.), which also led to more perceived hostilities towards Muslim minorities and more perceived discrimination (see e.g. Choudhury, Aziz, Izzidien, Khreeji, & Hussain, 2006 for the E.U.; Rousseau, Hassan, Moreau, & Thombs, 2011 for the United States). How does the extent to which immigrants from predominantly Muslim countries—and more specifically, the children of a very large and widely distributed immigrant group in Western Europe, namely the descendants of Turkish immigrants— themselves identify with their religion and their origins, and perceived discrimination, relate to their participation in local politics? In the political domain, immigrant voters, especially those immigrants and their children from predominantly Muslim countries, increasingly refrain from participating in local and national politics (Cesari, 2014; Just, Sandovici, & Listhaug, 2014; Michon & Vermeulen, 2013; Nielsen, 2013), even though political participation is a crucial vehicle for improving a group’s position in society (Bloemraad & Vermeulen, 2014).

Studies have researched what factors can explain differences between local voting turnout among native-born citizens, immigrants and the descendants of immigrants (e.g. Bevelander & Pendakur, 2011 for citizenship acquisition; Ruedin, 2018; Wass, Blais, Morin-Chassé, & Weide, 2015 for years since migration). Especially factors that relate to the act of migrating itself cannot provide us much insight into local voting turnout among the descendants of immigrants, even though the origins of their parents are still likely to affect their political participation, but in a different way. We have to look into factors that are related to the immigrant group, and not necessarily the migration experience. Ruedin (2018) researches factors such as the social origin (socio-economic status and resources), political engagement, networks and socialization, and provides a comprehensive overview of the literature.

How social identification relates to local voting is the focus of this study. Local voting is one important indicator of political participation, as it is basic to citizenship rights and a low-cost and little time-consuming manner of participating politically (Verba, Schlozman, & Brady, 1995). Researching immigrants’ descendants’ voting turnout is relevant because, among other things, Wass et al. (2015) assume that low participation often implies under-representation, which could lead to political exclusion, and any systematic exclusion of groups could reflect negatively on the functioning of a representative democracy (Wass et al., 2015). In general, most scholarly attention has been given to national elections (with exceptions e.g. Bevelander & Pendakur, 2011; Wass et al., 2015; Ruedin, 2018), even though turnout is often lower for local elections. This while the local level is where policies are most likely to be implemented and where they can affect the lives of individuals in serious ways (Hajnal & Lewis, 2003).

This study researches how some social psychological determinants relate to local voting, namely how the politicization of social identification is moderated by the interplay between perceptions of personal and group discrimination, focusing on Turkish and Islamic identifications among the descendants of Turkish immigrants. Perceived group discrimination and perceived personal discrimination are included separately as they refer to different processes and can be expected to affect the politicization of social identification differently.

The combinations of perceptions of personal and group discrimination can either enhance collective action or discourage it (Foster & Matheson, 1995, 1998, 1999). It could enhance collective action by making experiences of group discrimination personal, through direct encounters with personal discrimination. It would discourage mobilization if individuals became anxious due to this combination of personal and group experiences of discrimination and/or if it signaled stigmatization, which could lead to an anticipation of rejection of group interests by mainstream society (Fleischmann, Phalet, & Klein, 2011; Foster & Matheson, 1995, 1998, 1999). This study aims to contribute to this debate by appreciating how the various combinations of social identifications, and perceptions of group discrimination and personal discrimination, relate to political participation. The perception of both group discrimination and personal discrimination is expected to relate differently to political participation for individuals who identify more with the social group concerned. This study therefore wants to contribute to the existing debate by including the interplay between personal and group discrimination both theoretically and empirically in studying the politicization of social identification.

Scholars who have researched the politicization of social identification have sometimes neglected to distinguish between perceptions of personal and group discrimination, or have not considered how personal discrimination relates to political participation and collective action at all (e.g. Fischer-Neumann, 2014; Klandermans, 2014; Klandermans, Van der Toorn, & Van Stekelenburg, 2008; Kranendonk, Vermeulen, & Van Heelsum, 2018; Miller, Gurin, Gurin, & Malanchuk, 1981; Olsen, 1970; Simon & Klandermans, 2001). Alternatively, they did not account for the interplay between social identification, personal and group discrimination, and how this relates to political participation (Foster & Matheson, 1995, 1999; Schildkraut, 2005). This can lead to a distorted understanding of the politicization of social identification, since social identification’s combination with group discrimination is expected to relate differently to political participation in comparison to a combination with personal discrimination (e.g. Schildkraut, 2005; Smith, Pettigrew, Pippin, & Bialosiewicz, 2012; Walker & Pettigrew, 1984). Additionally, how the interplay between these three elements relates to political participation is also important to consider, since this interplay can either enhance political participation or discourage it (Foster & Matheson, 1995, 1998, 1999).

This study makes a threefold contribution to existing research. Firstly, it focuses on local voting, which is relevant due to elements mentioned before. Secondly, and unlike previous studies, it explores how the *interplay* between the three elements of social identification, perceptions of personal discrimination and perceptions of group discrimination relate to local voting. Thirdly, I acknowledge that the studied relationships can differ between country contexts, since countries’ policies concerning the political participation of immigrants can differ. The following research question is formulated:

*To what extent does the interplay between social identifications, perceptions of personal discrimination, perceptions of group discrimination, and country contexts relate to local voting likelihood among the descendants of Turkish immigrants in Western Europe?*

This study analyzes to what extent Turkish and Islamic identifications relate to the likelihood to vote in municipal elections among the descendants of Turkish immigrants in 10 Western European cities. Binary logistic regression analyses with clustered standard errors are used to study the cross-national survey from *The Integration of the European Second Generation* (TIES) project (Crul, Schneider, & Lelie, 2012; Fibbi, Topgül, Ugrina, & Wanner, 2015; Herzog-Punzenberger, 2010).^1^

## Conceptual framework

### Definitions

This study focuses on the descendants of Turkish immigrants who are defined as being born in the receiving country, but having one or two parents who were born in Turkey. This study will at times refer to ‘immigrants’ if this term is used by the literature that is discussed. I do relate to this literature because I expect that the immigrant origins can also affect the political participation of the children of immigrants. Furthermore, I refer to Muslim immigrants if this is mentioned in this way in the literature, and I refer to ‘Muslims’ in my analyses only when referring to the respondents who indicated to be religious themselves. The perception of group discrimination is defined as a perception of unfair group treatment and I define perceived personal discrimination as unfair treatment of an individual based on the individual’s perceived group membership (Schildkraut, 2005).

### Social identification and local voting

It is necessary to define social identification and explore how it relates to local voting, before elaborating on its interplay with personal and group discrimination. Social identification refers to individuals’ commitment to social categories, which is in turn expected to relate to the behavior of the individuals who belong to this group (Ellemers, Spears, & Doosje, 2002). Social identification concerns a dynamic process, which can vary over time and can be subjected to context (Brubaker & Cooper, 2000).

Social identification can relate to collective action through the process of depersonalization. Social identification puts emphasis on the group and leads to a *depersonalization* of the self-concept, which consists of a unique overlap of many social identities (Brewer, 1991; Turner, Hogg, Oakes, Reicher, & Wetherell, 1987). Turner et al. (1987) argue that depersonalization refers to how people come to see themselves less as unique individuals and more as an “interchangeable exemplar of a social category” (p. 50). They argue that depersonalization is the basic process that underlies group processes, such as collective action. Brewer (1991) explains that depersonalization relates to transformations in self-definitions, which can in turn alter “the meaning of self-interest and self-serving motivation” (p. 476). The social self can therefore also motivate individuals to act on behalf of the group instead of out of mere self-interest (Brewer, 1991). This relates to the perception of group interests, since social identification can increase the awareness of belonging to a group with shared political interests, which in turn enhances psychological engagement with politics and incentivizes political participation (Brady, Verba, & Schlozman, 1995).

Many forms of political participation can be used as an outlet of group behavior and as a vehicle for addressing the group’s position as well (e.g. see Kranendonk et al., 2018 for voting; Lee, 2008 for a theoretical overview; Olsen, 1970; Tate, 1991), even though the largest share of social psychological literature has theorized and researched collective action (e.g. Simon & Klandermans, 2001). Within the United States context, race and ethnicity have also been considered important dimensions in urban politics and local elections, even though empirical evidence on this has been mixed at times (see Hajnal & Trounstine, 2014 for an overview and empirical test). Dickson and Scheve (2006) argue that there is a considerable consensus that social identities (e.g. religious and ethnic groups) affect voting behavior and can structure voting choice by providing individuals with similar policy preferences. These preferences might not be represented by candidates or parties, which can lead individuals to refrain from voting. Therefore, social identification can relate to voting both positively and negatively, since individuals might not vote if none of the parties or politicians represent the group’s interests and its policy preferences (see also Kranendonk et al., 2018).

Turkish and Islamic identification, as forms of social identification, can therefore be theorized to relate positively to political participation (Kranendonk et al., 2018). Turkish and Islamic identification can however also be theorized to relate negatively to political participation (Kranendonk et al., 2018). Immigrants and their descendants who do not give up their origin-country identities could become segregated from mainstream society and refrain from participating in the political system. Religiosity can also facilitate traditional beliefs, religious principles and practices that can possibly complicate participation in the receiving society (Hirschman, 2004; Just et al., 2014).

Turkish and Islamic identification are also shown to relate to each other (see e.g. Verkuyten & Martinovic, 2012). Still, they can have separate effects on voting likelihood (Kranendonk et al., 2018). These identifications refer to different groups and can relate to various shared interests. The shared interests for ‘Turkish’ immigrants and their descendants (e.g. labor-market discrimination) do not necessarily overlap with shared interests for ‘Muslims’ (e.g. religious rights). Tiberj and Michon (2013, p. 286) also argue that ethnic minorities (in France) cannot be reduced to those who believe in Islam, since they argue that their religious composition is much more diverse (Brouard & Tiberj, 2005). Therefore it is important to consider how Turkish and Islamic identification relate to voting separately.

*H1a: Turkish identification relates to local voting likelihood among the descendants of Turkish immigrants in Western Europe.*

*H1b: Islamic identification relates to local voting likelihood among the descendants of Turkish immigrants in Western Europe.*

Considering the local context is crucial, seeing that local voting is the focus of this study. This calls attention to the supply side of local elections. Country and city characteristics such as the political opportunity structure, public discourse, citizenship regimes and institutional arrangements influence the opportunities that are offered to pursue immigrants’ and their descendants’ group-based interests. Koopmans, Statham, Giugni, and Passy (2005) show that citizenship regimes relate to the degree of claim-making on the basis of ethnicity and ethnic interests. Statham and Tillie (2016) provide a recent general overview of differences between countries according to cultural and religious pluralism, which can also relate to immigrants’ claim-making and political participation. Carol and Koopmans (2013) indicate that pre-existing church-state relations and citizenship rights regimes relate to the opportunities for religious claim-making.

How social identification relates to voting can depend on several contexts. Processes of voting turnout can differ between the local and national context. Cancela and Geys (2016) argue that there are at least two reasons for this. Firstly, they argue that there is clearly a discrepancy between engagement in national and local politics, and secondly, that voters may be situated in places (e.g. cities or neighborhoods) that accommodate specific processes of group mobilization that can enhance voting turnout in some elections, but not in others. How social identification relates to voting turnout on the local level could therefore differ with how it relates to the national level, which was the focus of previous studies (e.g. Kranendonk et al., 2018).

Concerning the national context, Koopmans (2004) argues that cross-national differences are still more important than local variations in explaining differences in migrant mobilization. Since I also expect country differences and not city differences in the extent to which social identification relates to local voting, country differences are discussed below.

It is important to acknowledge the specific domains of citizenship regimes and specific policies that are most likely to affect how immigrants’ and their descendants’ social identification relates to their local voting likelihood. The Migrant Integration Policy Index (MIPEX) gives the tools for this by providing scores for countries on various domains of integration policies. The domain which is coined ‘political participation’ seems most relevant for this study. It is composed of several dimensions, namely: electoral rights (to vote and to stand as a political candidate), political liberties (whether they are the same for immigrants as for national citizens), consultative bodies (whether immigrants can be consulted through consultative bodies), and implementation policies (whether immigrants get funding for political activities).^2^

The countries are given an aggregate score for the policy domain of political participation on the basis of their performance on these four dimensions, ranging from critically unfavorable (0 points) to favorable (80–100 points). Scoring lowest in 2007 and 2008, the years when the data used for this study was collected, is Austria (38 points), followed by France (52 points), Switzerland (58 points), Germany (61 points) and lastly the Netherlands (72 points).^3^ According to the MIPEX this corresponds to a slightly unfavorable context in this specific integration policy domain in Austria, a halfway favorable context in France and Switzerland and a slightly favorable context in Germany and the Netherlands.^4^ These integration policies surrounding the political participation of immigrants are of course not static and change over time. For example, between 2008 and 2014 Austria became more favorable in this specific policy domain, while the Netherlands became less favorable. It is however beyond the scope of this study to discuss or research the dynamic changes in integration policy domains.

The aforementioned country differences in policies concerning the political participation of immigrants are relevant since the politicization of social identification can also differ across contexts. Verkuyten (2016) shows that the perception of diversity ideologies moderates dual identification’s effects on intention to protest. He shows that dual identification’s effects on intention to protest are largest if individuals are exposed to multiculturalist diversity beliefs (versus assimilation). Turkish and Islamic identification probably relate more positively to local voting in contexts that provide favorable policies for the political participation of immigrants. These favorable contexts for the political participation of immigrants can enable immigrants and their descendants to mobilize in terms of their minority identities and to pursue immigrant or identity-related interests.

Since there are only ten cities included in this study, the hypothesis considering country differences is meant to be solely explorative, to see whether Turkish and Islamic identification relate positively to local voting in countries that have favorable policies for the political participation of immigrants and negatively in countries that are less favorable. The Netherlands is policy-wise the most favorable for the political participation of immigrants and Austria is the least favorable. Turkish and Islamic identification should then relate positively to local voting likelihood in the Netherlands, and negatively to voting likelihood in Austria, with the other countries in between.

*H2: Turkish and Islamic identification relate positively to local voting likelihood in countries that have more favorable policies for the political participation of immigrants (cities in the Netherlands) compared to countries that have less favorable policies (cities in Austria).*

### The politicization of social identification

Social identification by itself, however, doesn’t necessarily influence political participation, as it can also merely function as a point of self-reference (Lee, 2008; see also Miller et al., 1981; Simon & Klandermans, 2001). The perception of group discrimination can provide individuals with something to ‘fight’ for and is therefore an important element in studying the politicization of social identification (e.g. Kranendonk et al., 2018; Lee, 2008; Simon & Klandermans, 2001 who refer to shared grievances). Studying the interplay between social identifications, group discrimination and political participation without taking the perception of personal experiences of discrimination into account distorts our understanding of these relationships because they are expected to have different effects on political participation (see Walker & Pettigrew, 1984 and Smith et al., 2012 who refer to deprivation; Schildkraut, 2005). Additionally, the *interplay* between these elements, meaning the different combinations of levels of social identifications and perceptions of group and/or personal discrimination, can relate to political participation (Foster & Matheson, 1995, 1998, 1999). The following sections review the literature that deals with various aspects of, or related to, group discrimination and personal discrimination (e.g. grievances, stigmatization), social identification, and political participation, after which their interplay is addressed.

#### Group discrimination

Related to the concept of group discrimination is relative deprivation. The relationship between relative deprivation and collective action is well-researched. Scholars who focus on relative deprivation distinguish between egoistic (on the individual level) and collective deprivation (on the group level). Relative deprivation is defined as the unfavorable outcome of social comparisons, which are perceived to be illegitimate and stable (Walker & Pettigrew, 1984; see also Smith et al., 2012 for a theoretical overview and meta-analysis). Walker and Pettigrew (1984) give an overview of studies and argue that collective deprivation^5^ can enhance feelings of social injustice and motivate individuals to engage in collective action in order to change their deprived group status.

Simon and Klandermans (2001) argue that the perception of shared grievances, a concept which is also related to the perception of group discrimination, is an important element in the politicization of social identification. It can provide individuals with something to fight for, which can motivate mobilization on the basis of a social identity. Individuals are probably also more likely to be committed to improving the status of the group if they identify to a greater extent with the discriminated group. Various scholars have researched how social identification and perceptions of group discrimination (or related concepts) relate to political participation among immigrants and racial minorities. These scholars found positive correlations between identifications and political participation for those who were confronted with group discrimination or related phenomena (see also Kranendonk et al., 2018; Miller et al., 1981; Pérez, 2015 for negative political rhetoric).

Studies of the political participation of religious immigrant-origin groups found similar results for how a religious identity relates to political participation for individuals who are confronted with group discrimination. Just et al. (2014) find that especially among Muslim immigrants in Europe who have a negative attitude toward the host society, a reactive identity emerges as a way to deal with the discrimination and injustice they perceive. This reactive identity relates positively to political engagement among Muslim immigrants in an effort to create conditions that enable them to practice their beliefs.

*H3: Turkish and Islamic identifications relate positively to local voting likelihood if individuals perceive more group discrimination.*

#### Personal discrimination

In comparison to perception of group discrimination, there is less consensus about the existence and strength of the relationship between personal discrimination and political participation. Schildkraut (2005) theorizes that the perception of personal discrimination can also politicize social identification and suggests that personal discrimination makes individual self-concerns politically more powerful in comparison to group discrimination. However, Walker and Pettigrew argue there is neither theoretical grounding nor empirical support for a relationship between egoistic deprivation and social behavior (1984). Egoistic relative deprivation would relate to individual-serving attitudes and behavior (Smith et al., 2012), not group mobilization.

Foster and Matheson (1998) argue that egoistic deprivation is expected to relate to collective action if it is defined in relation to the out-group (I am deprived in comparison to members from a ‘different’ group) by raising consciousness and motivating collective action.

*H4: Turkish and Islamic identifications relate positively to local voting likelihood if individuals perceive more personal discrimination.*

#### Social identification, personal discrimination and group discrimination

The aim of this article is to include perceptions of personal experiences of discrimination into a unified framework to study the politicization of social identification. Personal discrimination is often neglected in research on the politicization of social identification (e.g. Klandermans, 2014; Klandermans et al., 2008; Kranendonk et al., 2018; Miller et al., 1981; Olsen, 1970; Simon & Klandermans, 2001). Scholars who have considered the effects of both the perceptions of group and personal discrimination did not take into account the extent to which individuals identify with social categories (Foster & Matheson, 1995, 1999; with the exception of Schildkraut, 2005 who refers to self-identification in terms of American, pan-ethnic or Latino, but not the *extent* of identification with these categories). Lastly, scholars who considered social identification, personal and group discrimination did not take into account that the *interplay* between these three elements matters for how social identification relates to political participation, or that the *extent* of social identification matters (Schildkraut, 2005).

The interplay between the perception of group and personal discrimination can relate to political participation both positively and negatively. Foster and Matheson (1995, 1998, 1999) argue that the combination of personal and group discrimination can mobilize individuals for collective action, which could be used to address perceived discrimination. If individuals also experience personal discrimination on the basis of the social category they identify with, their experiences become integrated with the group’s experiences. In other words, the individuals’ problems will overlap the group’s problems (Foster & Matheson, 1995, 1998). Collective action in order to address group discrimination will then also be seen as personally relevant for one’s individual status, thus the combination between perceived personal and group discrimination can motivate mobilization (see also Foster & Matheson, 1995, 1999 for empirical examples for women).

Social identification combined with personal discrimination as well as perceptions of group discrimination can also relate negatively to collective action. Fleischmann et al. (2011) argue that the perception of discrimination may also depoliticize social identity. They give the empirical example of support for political Islam and willingness to engage in political action among Turkish and Moroccan Muslims in Europe, and argue that personal discrimination on the basis of their religious background may signal stigmatization of Muslims by the European majority population. Depending on the extent that personal discrimination signals stigma, Muslims may refrain from mobilizing around a stigmatized religious identity because they might anticipate rejection by the dominating majority population of political claims that are based on their religious identity (p. 631). Deriving from the theoretical framework of *double* discrimination (Foster & Matheson, 1995, 1998, 1999) it can be expected that the perception of group discrimination, next to personal discrimination, also matters for the politicization of social identification. Additionally, it could be expected that discrimination is most likely to signal stigma if individuals feel that their group is also discriminated against, next to personal experiences of discrimination.

If individuals perceive that ‘their’ social group is discriminated against, and they are themselves affected by discrimination on the basis of this category, recognizing the extent to which society can affect their personal lives might make them anxious (Crosby, 1984; Foster & Matheson, 1995, 1999). The perception of both personal and group discrimination might be accompanied by strong negative emotions, which can impair behavior such as collective action (Foster & Matheson, 1998) or political participation. These strong negative emotions, such as anxiety and depression, could lead individuals to focus more on managing these emotions and distract them from attempting to improve their group’s status (Foster & Matheson, 1998).

**Fig. 1:**
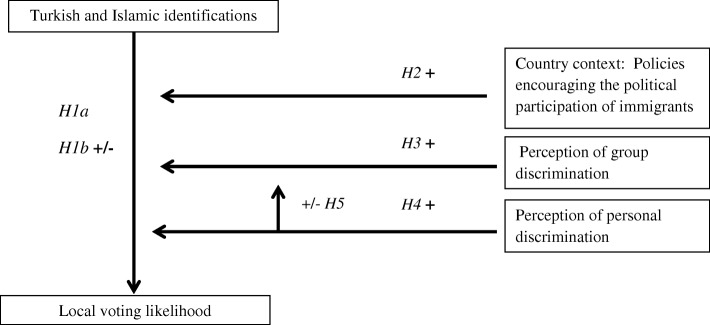
Hypotheses. It shows the main concepts of the article (e.g. Turkish identification), the presumed direction of the effects and the moderators (e.g. perception of group discrimination)

The extent to which the interplay between perceptions of group and personal discrimination relates to political behavior can vary according to individuals’ self-identifications. These mechanisms are expected to work differently for low identifiers in comparison to high identifiers, as is also theorized for the relationship between social identification, group discrimination and collective action (e.g. Simon & Klandermans, 2001). Based on these theories I take into consideration that the combination of social identification, and perceptions of both personal and group discrimination, could relate positively and negatively to local voting likelihood (hypothesis 5) (Fig. 1).

*H5: Turkish and Islamic identifications, in combination with the perception of group discrimination and personal discrimination, relate to local voting likelihood.*

## Data and methods

The TIES surveys (conducted in 2007 and 2008) are used for this article. The large-scale TIES (The Integration of the European Second Generation) project conducted surveys in fifteen European cities, in eight countries, in order to research the integration of descendants of Turkish, Moroccan and former Yugoslavian immigrants. The TIES project defined the second generation as individuals who are born in the receiving country, have at least one parent who was born in the origin country and who followed their education in the receiving country.^6^ Their target population was between 16 and 35 years old and the interviews were conducted in the receiving country’s language (e.g. Dutch in the Netherlands) (for more information: Crul et al., 2012; Fibbi et al., 2015; Groenewold & Lessard-Phillips, 2012; Herzog-Punzenberger, 2010).

This study limits its analyses to the Turkish second generation in order to maintain a more homogenous sample. Turkish immigrants and their children are a large immigrant group in many Western European countries. According to Crul, Schneider, and Lelie (2013) the descendants of Turkish immigrants are a very suitable group for international comparison, since they share cultural and geographical characteristics, as well as a similar socio-economic starting position, namely born to parents with low educational levels (mostly guest workers). However, some studies found differences between rural and urban origins (e.g. Lancee & Seibel, 2014) and argue that there is great polarization between secular and religious groups and between religious denominations (Vermeulen, 2013). Control variables are added to the model in order to attempt to account for these variations.

The selection of the descendants of Turkish immigrants resulted in a sample of ten cities in five countries, which depended on the data availability (e.g. only Moroccan individuals were surveyed in Spain) (Crul et al., 2012) and particularities within the selected countries. The latter refers to compulsory voting in Belgium, which resulted in very high scores on the probability to vote, my dependent variable. Whether someone then votes because of compulsory voting (though it is not enforced anymore) or because of one’s identity is not clear, and therefore Belgium was omitted from the analyses. The surveys from Austria, France, Germany, Netherlands, and Switzerland are used. Only the respondents who have citizenship of the receiving country are included in the analyses, with the exception of 13 respondents in the Netherlands.^7^ Here residents can vote after having lived in the Netherlands for 5 years, which is the case for the descendants of immigrants.

Religious and non-religious respondents are included in order to research the association between Turkish identification and local voting (1627 respondents). I include non-religious individuals since they can still identify as Turkish and also pursue Turkish group interests via local voting. Excluding the non-religious individuals gives distorted insights into the relationship between Turkish identification and local voting, and it is problematic to generalize to the descendants of Turkish immigrants in Western Europe, because not all of them are Muslim—as also reflected in this sample, within which 398 individuals indicated that they are not Muslim. Furthermore, this is in line with other studies that point out the religious diversity among immigrant-origin groups (see e.g. Brouard & Tiberj, 2005 for France). Non-Muslim individuals are excluded in order to explore correlations between Islamic identification and local voting (1229 respondents).

Binary logistic regression analyses are used in order to deal with the non-linearity and non-normality of the distribution of errors of the dependent variable. I include fixed effects for countries and I include standard errors that are clustered at the city level, which accounts for the nested nature of the data. The results are presented in log odds, where smaller than zero represents a negative relative effect and larger than zero represents a relative positive effect. The graphical displays show the marginal effects and I also include tables that show the differences between the predicted probabilities to vote for the highest identifiers versus the lowest identifiers.

Cross-sectional research can always suffer from omitted variable bias. The selection of citizens and the role of the parents could cause this. Individuals who obtained citizenship are probably more likely to vote, more likely to identify with the receiving country and less likely to identify with the origin country. The association between national identification and local voting can therefore be overestimated due to the selection of citizens, though this is not the main focus of this study. If citizenship increased the likelihood to vote, and decreased Turkish identification, then the selection of solely citizens could make this association look more negative, or less positive, than it would be for non-citizens. Since the focus of this study is on local voting, which in many countries requires citizenship, this is not very problematic. It is more problematic if we want to generalize these findings to other types of political participation, which do not require citizenship. Turkish and Islamic identification could be more positively related to these types of political participation in comparison to (local) voting. Parents also influence their children in many ways (e.g. educational attainment) and can influence their children’s identification (e.g. Hughes et al., 2006), as well as their political participation (e.g. Spierings, 2016). The lack of this information about the parents could lead to an overestimation of the association between identifications and voting likelihood. However, it is still interesting to look at how these individual identifications affect voting likelihood, regardless of how this association came into being (e.g. partly socialized by the parents), since these identifications seem to be related to adult behavior. Analyzing the role of the parents could be interesting for future studies.

Using self-reported voting as a dependent variable has a few drawbacks, including possible over-reporting due to social desirability, the over-sampling of voters in surveys (Sciarini & Goldberg, 2016), and differences between cities due to some elections being longer ago than others. Furthermore, the perception of suitable political candidates and political parties can influence the relationship between Turkish and Islamic identifications and local voting (e.g. see Simon & Klandermans, 2001 about the role of leaders, and Dickson & Scheve, 2006 for group-based appeals). Unfortunately, the data does not allow me to research this specific mechanism.

Another drawback of the data concerns its cross-sectional nature, which does not allow me to control for issues of causality. From the results shown it cannot be derived whether social identifications precede local voting, even though this is theoretically expected.

### Dependent variable

The respondents were asked whether they voted during the last municipal elections. The respondents who stated that they did not have voting rights were deleted from the sample. In total 909 respondents indicated to have voted (56%) and 718 respondents indicated not to have voted (44%). Among the religious sample 672 individuals indicated to have voted (55%) and 557 respondents indicated not to have voted (45%). The table below shows the distribution of the sample according to voting and countries (Table 1).

**Table 1:** Municipality voting distribution of respondents according to country

*Cities*	Total sample (including non-religious)			Religious sample		
	Not voted	Voted	Total (100)%	Not voted	Voted	Total (100%)
Austria	46.4	53.6	334	52.2	47.8	251
France	42.7	57.3	342	42.1	57.9	304
Germany	55.0	45.0	353	62.8	37.2	218
Switzerland	44.2	55.8	249	43.3	56.7	150
The Netherlands	32.4	67.6	349	31.4	68.6	306
Total	44.1	55.9	1627	45.3	54.7	1229

Source: TIES surveys 2007/2008

### Independent variables

Turkish identification is operationalized with the question ‘To what extent do you feel… Turkish?’ and Islamic identification is operationalized with the question relating to feeling ‘Muslim’ (1 ‘Very weakly’ to 5 ‘Very strongly’). Many individuals who indicated not to be religious did answer the question about the extent they felt Muslim, except in the Netherlands. These scores could show an attachment to Islam beyond religious experiences, such as cultural attachments. The missing values for the 35 non-religious respondents in the Netherlands were given the mean value of Islamic identification of the non-religious respondents in the other countries. The respondents with another religion were given the lowest value on the variable that inquires about feeling Muslim. The non-religious respondents in the Netherlands and Christians in general were also modeled as a dummy in order to limit their influence in the analyses. This modeling strategy has the downside that it assumes a homogeneity among the respondents who are modeled as missing, namely that they are not Muslim. However, it also enables me to control for Islamic identification in order to estimate correlations for Turkish identification, which is more valid since the secular descendants of Turkish immigrants can identify with being Turkish without having to be religious. Analyzing solely the Muslim descendants of Turkish immigrants would contribute to the discourse of discussing immigrants and their children from predominantly Muslim countries solely in terms of their religion, whether they are religious or not. The results for Turkish identification are similar, with a minor exception (see robustness checks) if the non-Muslim respondents (398) are excluded from the analyses, which indicates that the modeling strategy for the missing values does not affect the main results. The non-religious respondents and Christian respondents (398) are excluded from the analyses for Islamic identification and local voting.

The perception of group discrimination is operationalized as: ‘In general, how often do you think that the following groups experience hostility or unfair treatment because of their origin or background…?’ in the concerning countries? This question was asked with regard to the Turkish population (perception of Turkish group discrimination) and Muslims (perception of group discrimination of Muslims), with answers between 1 (‘never’) and 5 (‘frequently’). Personal discrimination is operationalized with the question: ‘Have you ever experienced hostility or unfair treatment towards you because of your origin or background, either as a child or later in life?’ (1 ‘Never’ to 5 ‘Frequently’).

Turkish and Islamic identification and perceived discrimination could, but do not necessarily have to, relate. Various pieces of research argue that discrimination can affect identification, or the other way around (e.g. Branscombe, Schmitt, & Harvey, 1999). Nevertheless, social identification, referring to feelings of connectedness with the in-group, differs conceptually from perceived treatment by an out-group of the self (personal discrimination) or the in-group (group discrimination) based on group membership. However, researching this in depth is beyond the scope of this study and I will solely reflect on the correlations between my included main variables. Both forms of identification do not relate statistically significantly to perceived group discrimination, or to perceived personal discrimination. The perceived group discrimination of Turkish individuals and Muslims relates positively to perceived personal discrimination (.27 and .38 as correlation measures that are both statistically significant with *P* < .001). Even though these forms of perceived discrimination correlate, the association shows that there is still some discrepancy between the forms of discrimination (Foster & Matheson, 1999).

### Control variables

Just as is the case with non-immigrant individuals, voter turnout among the descendants of immigrants can, to a large extent, be explained by ‘general’ turnout-related indicators such as educational attainment (Verba et al., 1995).

Gender, age, educational level (primary, secondary or tertiary), hours worked per week and monthly income (in nine categories) are included. The number of hours worked per week and monthly income generated quite a few missing values which were also modeled as a dummy in order to eliminate their influence without deleting them from the analyses.

The exposure to recruitment networks and the composition of networks are also acknowledged to relate to voter turnout (Verba et al., 1995). In order to be able to control for personal networks, questions about the national origin of the best friend (native, Turkish or other background) and the composition of the neighborhood were asked. The latter question asked respondents to describe their neighborhood in terms of national-origin composition (1 ‘A neighborhood where almost nobody is of Turkish origin’, to 5 ‘A neighborhood where almost everyone is of Turkish origin’).

There are also some variables included that relate more specifically to the Turkish and religious background of the respondents. Religious affiliation is distinguished between Sunna, Shia, Alevi, other Muslims and non-believers. Language proficiency of the survey countries’ languages is measured with a scale indicating how well the respondents speak, read and write the survey country’s language (1 ‘bad’, to 6 ‘excellent’). The three items for language proficiency form a very reliable scale (Cronbach’s α > .90). National identification (identification with the receiving country) is operationalized with the question ‘To what extent do you feel… National?’^8^ (1 ‘Very weakly’ – 5 ‘Very strongly’).

## Results

Firstly, the associations between Turkish, Islamic identification and local voting likelihood are estimated, as well as their differences between countries. After this, interactions of Turkish and Islamic identifications and perceptions of group discrimination and personal discrimination are estimated. The interactions are presented in figures that are based on the tables in the Appendix.

Table 2 shows that Turkish and Islamic identifications do not relate *similarly* to local voting across the included countries, thus I find no support for hypotheses 1a and 1b.

**Table 2:** Effects on local voting likelihood

	Model 1	Model 2
Social Identification		
Turkish identification	.09 (.09)	.20 (.11)†
Islamic identification	.02 (.06)	.04 (.08)
Perceived Discrimination		
Group discrimination of Turkish-origin individuals	.07 (.09)	.06 (.07)
Group discrimination of Muslims	−.08 (.08)	−.09 (.08)
Personal experiences of discrimination	−.02 (.07)	.04 (.06)
Control variables		
Age	.07 (.02)***	.08 (.02)***
Women (ref. men)	.05 (.12)	−.02 (.12)
Middle education (ref. lower)	−.30 (.40)	−.19 (.47)
Higher education (ref. lower)	.24 (.51)	.33 (.63)
Hours worked per week	.00 (.01)	.01 (.01)
Monthly income	−.04 (.06)	−.08 (.06)
Language proficiency	.12 (.03)***	.11 (.04)**
National identification	.25 (.11)*	.21 (.10)*
National-origin composition neighborhood	−.04 (.07)	−.02 (.07)
Turkish best friend (ref. native best friend)	−.58 (.19)**	−.36 (.21)†
Other origin best friend (ref. native best friend)	−.17 (.21)	.13 (.21)
Islamic denomination (ref. Sunni)		
Shia	.57 (.38)	.74 (.43)†
Alevi	.38 (.28)	.45 (.29)
Other Muslims	−.10 (.10)	−.10 (.10)
Non-believer	.22 (.17)	–
Countries (ref. Austria)		
France	.00 (.33)	.14 (.27)
Germany	−.36 (.41)	−.56 (.35)
Switzerland	−.01 (.49)	.19 (.44)
The Netherlands	.91 (.34)**	.94 (.30)**
N	1627	1229
Df	29	28
Prob>Chi2	.000	.000

The results are reported in log odds, the standard errors are indicated in parentheses and the standard errors are clustered on cities

Source: TIES survey 2007/2008; † *p* < 0.10 *p < 0.05 **p < 0.01 ***p < 0.001

**Fig. 2:**
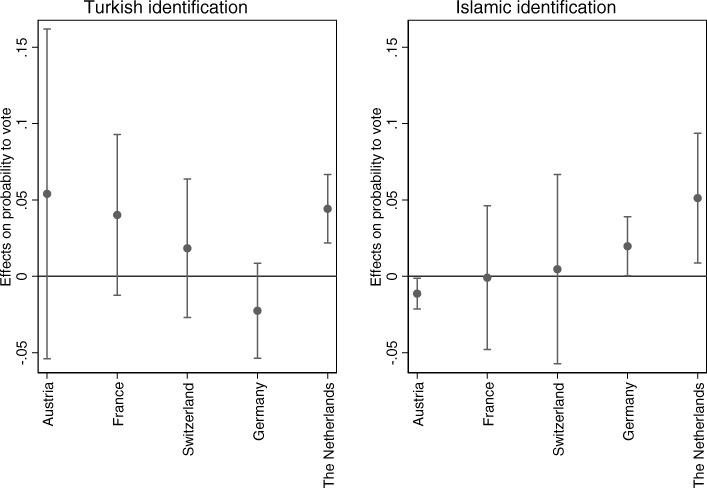
Effects of Turkish and Islamic identifications on local voting according to country (TIES surveys 2007/2008). These graphs show the marginal effects of Turkish and Islamic identification on the probability to vote locally (vertical axis) for different countries (horizontal axis)

Figure 2 (Table 9 in Appendix) shows that Turkish and Islamic identifications relate to local voting if the differences between countries are taken into account (hypothesis 2). Turkish identification relates positively to voting probability among the descendants of Turkish immigrants living in Austria, France, Switzerland and the Netherlands, even though only the latter association is statistically significant. High Turkish identifiers are 18 percentage points more likely to vote in the Netherlands compared to low Turkish identifiers (see Table 3). Turkish identification relates negatively to voting probability among those living in Germany, though not statistically significantly (*N* = 1627). Islamic identification relates positively to local voting likelihood among the religious voters living in Germany and the Netherlands while it relates negatively to voting among those living in Austria. High Islamic identifiers are eight percentage points more likely to vote in comparison to low Islamic identifiers in Germany and 21 percentage points more likely in the Netherlands, while in Austria high Islamic identifiers are five percentage points less likely to vote in comparison to low identifiers (Table 3). Islamic identification does not relate to voting likelihood among the religious descendants of Turkish immigrants living in France and Switzerland (*N* = 1229).

**Table 3:** Differences in the predicted probabilities between the highest identifiers and lowest identifiers, according to country

	Austria	France	Switzerland	Germany	The Netherlands
Difference Turkish high identifier-low identifier	0.21	0.16	0.07	−0.09	0.18
Difference Islamic high identifier-low identifier	−0.05	−0.01	0.01	0.08	0.21

For voters that live in the country that provides the most encouraging policies to enhance immigrants’ political participation (the Netherlands), Turkish and Islamic identifications relate positively to local voting likelihood. This corresponds to the theoretical expectations. These expectations are best reflected for Islamic identification, which relates negatively to voting in the country that has the least encouraging policies, namely Austria, while Islamic identification relates positively to voting in countries with the more encouraging policies, namely Germany and the Netherlands. The pattern is less clear when it comes to Turkish identification, since it relates positively to voting in Austria (though not significantly) and in the Netherlands, the countries that differ the most when it comes to policies surrounding the political participation of immigrants. Therefore only partial support for hypothesis 2 is found.

### The perception of group discrimination and personal discrimination

**Fig. 3:**
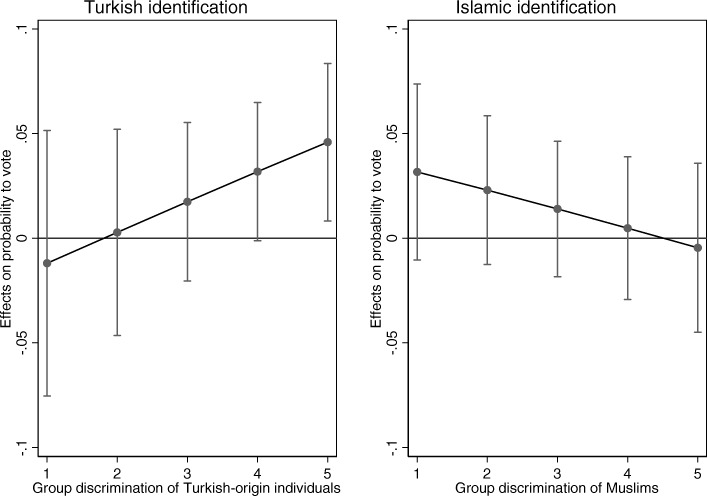
Effects of Turkish and Islamic identifications on local voting according to perceptions of group discrimination (TIES surveys 2007/2008). These graphs show the marginal effects of Turkish and Islamic identification on the probability to vote locally (vertical axis) for different levels of perceived group discrimination of Turks or Muslims (horizontal axis)

Figure 3 (Table 10 in Appendix) shows that Turkish identification relates positively to voting likelihood for individuals who perceive more group discrimination. High Turkish identifiers are 18 percentage points more likely to vote in comparison to low Turkish identifiers when both these groups perceive many instances of group discrimination of Turkish individuals. High identifiers are somewhat less likely to vote (5 percentage points) in comparison to low identifiers if these groups perceive no group discrimination (see Table 4). Islamic identifications show a different trend and does not relate statistically significantly to voting, regardless of perceived group discrimination of Muslims. Therefore, partial support for hypothesis 3 is found, but only for Turkish identification.

**Table 4:** Differences in the predicted probabilities between the highest identifiers and lowest identifiers, according to perceived group discrimination

	Perceives little group discrimination	Perceives a lot of group discrimination
Difference Turkish high identifier-low identifier	−0.05	0.18
Difference Islamic high identifier-low identifier	0.13	−0.02

**Fig. 4:**
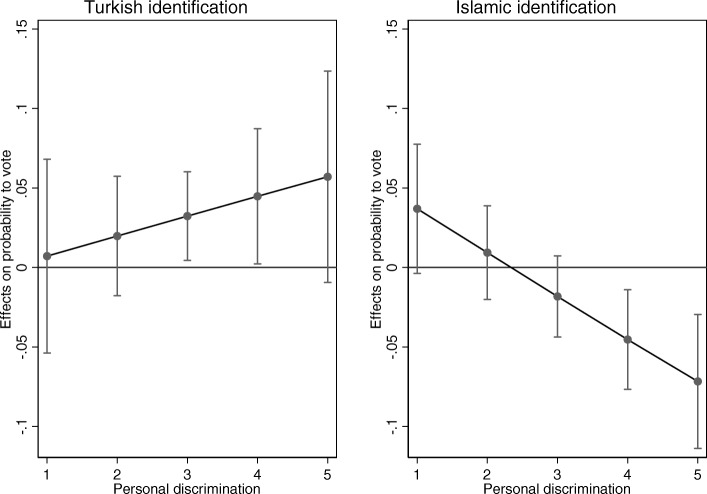
Effects of Turkish and Islamic identifications on local voting according to perceptions of personal discrimination (TIES surveys 2007/2008). These graphs show the marginal effects of Turkish and Islamic identification on the probability to vote locally (vertical axis) for different levels of perceived personal discrimination (horizontal axis)

Figure 4 (Table 11 in Appendix) shows the results of the interactions between Turkish and Islamic identification and perceptions of personal discrimination. Turkish identification relates positively to local voting likelihood if individuals perceive more discrimination (*P* < .05 one-tailed test), similarly to when they perceive more group discrimination (Fig. 3). High Turkish identifiers are 23 percentage points more likely to vote in comparison to low Turkish identifiers if both groups perceive many instances of personal discrimination (Table 5). Islamic identification relates negatively to voting likelihood if individuals perceive more personal discrimination. High Islamic identifiers are 26 percentage points less likely to vote in comparison to low Islamic identifiers if both groups perceive many instances of personal discrimination (Table 5). This is in line with studies that argue that personal experiences of discrimination make individual self-concerns more politically powerful in comparison to group experiences (Schildkraut, 2005). However, I find support for a negative relationship for Islamic identification and the hypothesized positive relationship for Turkish identification (hypothesis 4).

**Table 5:** Differences in the predicted probabilities between the highest identifiers and lowest identifiers, according to perceived personal discrimination

	Perceives little personal discrimination	Perceives a lot of personal discrimination
Difference Turkish high identifier-low identifier	0.03	0.23
Difference Islamic high identifier-low identifier	0.15	− 0.26

Social identification, combined with the perception of group discrimination and personal discrimination, can relate both positively and negatively to local voting. However, the interaction effects of Turkish identification and the different combinations of personal and group discrimination on local voting likelihood are not statistically significant (Hypothesis H5: Table 12 in Appendix).

**Fig. 5:**
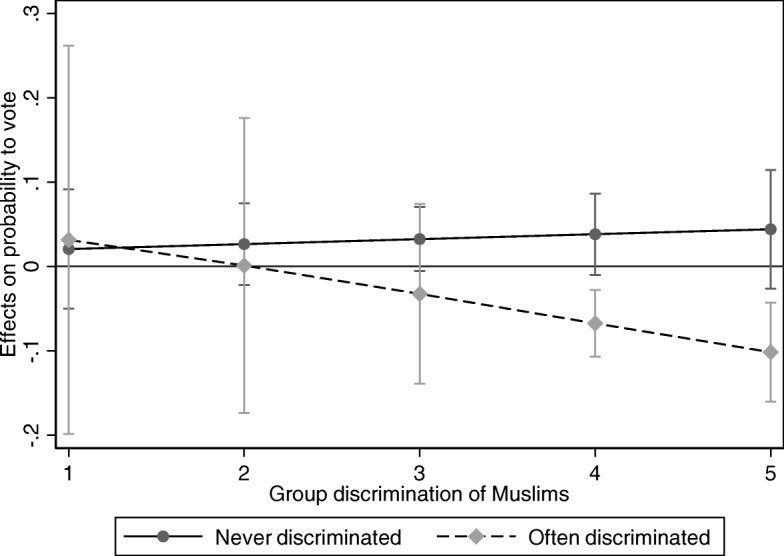
Effects of Islamic identifications on local voting for different combinations of the perception of group discrimination and the perception of personal discrimination (TIES surveys 2007/2008). This graph shows the marginal effect of Islamic identification on the probability to vote locally (vertical axis) for different levels of perceived group discrimination of Muslims (horizontal axis) and levels of perceived personal discrimination. The solid line represents the respondents who never feel discriminated, while the dashed line represents the respondents who often feel discriminated

Concerning Islamic identification, Fig. 5 (Table 12 in Appendix) shows that Islamic identification relates positively (though not significantly) to local voting likelihood for individuals who hardly perceive any group discrimination and never, or often, experience personal discrimination (*N* = 1229). It also relates positively, though not significantly, to voting likelihood among individuals who experience many instances of group discrimination and never experience personal discrimination. Islamic identification only relates negatively to local voting if individuals perceive more group discrimination of Muslims and often experience personal discrimination. Among the group that perceives both many instances of group and personal discrimination, Islamic high identifiers are 37 percentage points less likely to vote in comparison to Islamic low identifiers. Table 6 further shows that high Islamic identifiers are more likely to vote in comparison to low Islamic identifiers for the other combinations of perceived personal and group discrimination. This finding can be explained by either the anxiety mechanism - the combination of group discrimination and personal discrimination could be anxiety-provoking (Crosby, 1984; Foster & Matheson, 1995, 1998, 1999) - or the stigmatization mechanism, which can lead to an anticipation of rejection of religious claims by mainstream society (Fleischmann et al., 2011), which is probably more likely for individuals who identify with being Muslim and perceive both group and personal discrimination. Concerning Islamic identification, this provides support for hypothesis 5.

**Table 6:** Differences in the predicted probabilities between the highest identifiers and lowest identifiers, according to the lowest and highest scores on perceived group and personal discrimination

	Low group discrimination & low personal discrimination	Low group discrimination & high personal discrimination	High group discrimination & low personal discrimination	High group discrimination & high personal discrimination
Difference Turkish high identifier-low identifier	−0.07	0.17	0.15	0.20
Difference Islamic high identifier-low identifier	0.08	0.14	0.17	−0.37

### Robustness checks

Interacting the interaction effects (H3, H4 and H5) with countries is too demanding for the statistical models. The interactions could however differ between countries according to their policies regarding the political participation of immigrants. The analyses were therefore also conducted excluding one country at a time. Except for the main effects of Turkish and Islamic identification, as already shown in Fig. 2, the results of the interaction effects are similar and are therefore not driven by one particular country. The interactions between the identifications and perceived group and/or perceived personal discrimination (H3, H4, H5) were also similar if city-fixed effects were included in the models instead of country-fixed effects.

The association between Turkish identification and local voting was also analyzed excluding the non-Muslim respondents (398). The results are mainly similar (there are some small changes in the significance levels due to the decreased sample size), with one exception in terms of the association between Turkish identification and local voting according to country (Fig. 2). Including the non-Muslim respondents, Turkish identification relates negatively, though not significantly, to local voting in Germany. Excluding the non-Muslim respondents, Turkish identification relates positively and significantly to local voting likelihood. Maybe the mobilization according to Turkish background intersects with religion to such an extent in Germany that Turkish identification only relates positively to voting among religious voters. This is however an empirical puzzle and beyond the scope of this research to explore in depth.

## Conclusion

This study shows that Turkish and Islamic identifications relate to local voting likelihood among the descendants of Turkish immigrants in Western Europe, but only under specific circumstances.

Turkish and Islamic identifications were expected to relate positively to local voting likelihood in countries with more policies to encourage the political participation of immigrants (MIPEX scores on political participation). Country policies that encourage the political participation of immigrants seem to encourage the mobilization of both Islamic and Turkish identities, but the pattern for Turkish identification is less clear.

Turkish identification also relates positively to local voting likelihood for voters who perceive more group discrimination, and for voters who perceive more personal discrimination. This is in line with the theoretical expectations (e.g. Kranendonk et al., 2018; Miller et al., 1981) and corresponds with the idea that group discrimination can provide individuals with something to fight for, which can motivate mobilization (Simon & Klandermans, 2001). For Islamic identification there are other patterns. Islamic identification does not relate statistically significantly or negatively to voting likelihood for voters who perceive many instances of group discrimination, or voters who perceive many instances of personal discrimination.

In line with Foster and Matheson (1995, 1998, 1999) I find that both the perception of group and personal discrimination matters for the relationship between social identification and political participation. Islamic identification relates negatively to voting likelihood among religious descendants of Turkish immigrants who perceive more group discrimination of Muslims as well as personal discrimination. This negative relationship could be explained by the anxiety mechanism, namely that the combination of group and personal discrimination provokes anxiety (Crosby, 1984; Foster & Matheson, 1995, 1999). Strong negative emotions can distract individuals from attempting to improve the group’s position (Foster & Matheson, 1998). Secondly, double discrimination, as well as connectedness to the discriminated group, might signal stigma. Perceptions of stigmatization can make individuals anticipate rejection of their pursuit of group-based interests (Fleischmann et al., 2011).

Seeing that voting is a low-cost manner of participating locally, and basic to citizenship rights (Verba et al., 1995), withdrawal from local politics can have serious effects. It could lead to more alienation from Western European politics. Low participation can reflect under-representation, which could result in political exclusion. The systematic exclusion of certain groups, in this case high identifier Muslims, can reflect poorly on the functioning of representative democracies (Wass et al., 2015).

What do these findings teach us? Starting with research on the politicization of social identification: Turkish and Islamic identifications relate to local voting likelihood among the descendants of Turkish immigrants in Western Europe. In researching the politicization of social identification, the interplay between social identification, the perceptions of group discrimination, personal discrimination and the policy context should be taken into account, since they can have interactive effects on political participation. This study also shows that Turkish and Islamic identifications can relate differently to local voting, depending on the circumstances (e.g. perceived personal discrimination). This underlines the importance of *not* collapsing origin country and religious categories, as is becoming increasing usual when it comes to immigrants and their descendants from predominantly Muslim countries. Not only are these groups religiously diverse (see e.g. Brouard & Tiberj, 2005 for a French example) but the commitment to these identities can also relate differently to political behavior (see also Kranendonk et al., 2018).

Future studies could focus on whether these findings also hold if we consider other forms of political participation (see data and methods section) and whether this can be generalized to other groups. The size and settlement pattern of Turkish immigrants in Europe make it worth studying the political behavior of their children by itself. The theoretical substantiation for the association between social identification and local voting is not particular to the descendants of Turkish immigrants, nor are the interaction effects that I studied, which means that these mechanisms could also work for other immigrant-origin groups. However, the Turkish group is specific in the sense that they identify highly with their Turkish origins, and are very well politically organized, while they are also polarized (e.g. Vermeulen, 2013). They might be better able to mobilize around a social identity because of better political organization, but on the other hand this might be challenging because of polarization. Furthermore, the descendants of Turkish immigrants are an identifiable immigrant-origin group, which could also make them more vulnerable for discrimination in comparison to immigrant-origin groups that are not identifiable. In the end, the generalization of these findings to other immigrant-origin groups is an empirical question that requires more research.

Research can also focus more on specific local-level factors that can influence the politicization of a social identity of descendants of immigrants at the local level. Cancela and Geys (2016) mention some expectations at the context level, such as group size, concentration and party systems, that can affect aggregate voting turnout. Maybe these contextual characteristics also affect the politicized identity? Party level factors, such as whether someone of Turkish origin stands as a candidate, could also motivate high Turkish identifiers to vote. Some studies indeed show that some groups of voters vote more often for a candidate with a similar immigrant origin (Fisher et al., 2015).

In terms of policy making, these findings teach us that immigrants and their descendants should feel that they can address group interests by participating in local politics. If they feel that these group-based claims or their identities are rejected, it is possible that they will increasingly feel alienated from the local political system and refrain from participation. However, one could also argue that the system for this is already in place and that the groups that participate less are in some way less able, or unwilling, to mobilize. Future research focusing on the perceptions of immigrants and their children on the usefulness of political participation for pursuing group-based interests could provide us more insights into these specific mechanisms. It is relevant to study their local voting turnout as it is a crucial vehicle to influence local policy-making. It is the level where policies are most likely to be implemented (Hajnal & Lewis, 2003), and the political level that can affect lives in serious ways (e.g. Hajnal & Lewis, 2003). Regardless of whether the integration policies caused it, or whether it is due to factors related to the concerned groups, lower participation (in this case from high Islamic identifier descendants of Turkish immigrants) can lead to under-representation, which in turn can lead to political exclusion. Political exclusion in turn can reflect poorly on the functioning of representative democracies (Wass et al., 2015).

## Notes

1. The TIES Switzerland data was made available by the Principal Investigator Rosita Fibbi.
2. The Austrian data were made available by the Principal Investigator Barbara Herzog-Punzenberger.
3. The German TIES data were made available by the Principal Investigators Andreas Pott and Jens Schneider.
4. The Dutch TIES data were made available by the Principal Investigator Maurice Crul.
5. The French TIES data were made available by the Principal Investigator Patrick Simon.

## Appendix

**Table 7:** Descriptive variables

	Total sample		Religious sample		Min	Max
	Mean	St. dev.	Mean	St. dev.		
Age	24.8	4.8	24.6	4.7	18	35
Language proficiency	15.7	2.6	15.5	2.6	3	18
Hours worked per week	36.9	6.9	36.5	6.7	12	60
Monthly income	2.9	1.5	2.8	1.4	1 (Less than 550 €)	9 (More than 5000 €)
National-origin composition neighborhood	2.2	1.0	2.3	1.0	1 (No Turkish residents)	5 (Mostly Turkish residents)
National identification	3.3	1.0	3.2	1.0	1 (Very strongly)	5 (Very weakly)
Turkish identification	3.9	1.1	4.1	1.0	1 (Very strongly)	5 (Very weakly)
Islamic identification	3.7	1.3	4.2	1.0	1 (Very strongly)	5 (Very weakly)
Perceived group discrimination of Turkish-origin individuals	3.1	1.1	3.1	1.1	1 (Never)	5 (Frequently)
Perceived group discrimination of Muslims	3.6	1.2	3.6	1.2	1 (Never)	5 (Frequently)
Personal discrimination	2.0	1.0	2.0	1.0	1 (Never)	5 (Frequently)
N	1627		1229			

Source: TIES surveys 2007/2008

**Table 8:** Categorical variables

	Total sample		Religious sample	
	Count	Freq.	Count	Freq.
Voted during last municipality elections				
Yes	909	55.9	672	54.7
No	718	44.1	557	45.3
Gender				
Men	767	47.1	582	47.4
Women	860	52.9	647	52.6
Education				
Primary	65	4.0	58	4.7
Secondary	1215	74.7	930	75.7
Tertiary	347	21.3	241	19.6
National origin best friend				
Native	457	28.1	267	21.7
Turkish	985	60.5	822	66.9
Other	167	10.3	123	10.0
Islamic denomination				
Sunnite	867	53.3	867	70.6
Shiite	38	2.3	38	3.1
Alevi	98	6.0	98	8.0
Other Muslims	226	13.9	226	18.4
Other/Non-believer	398	24.5	–	–
Missing				
Hours worked per week	659	40.5	540	43.9
Monthly income	427	26.2	315	25.6
National origin best friend	18	1.1	17	1.4
National-origin composition neighborhood	62	3.8	39	3.2
Cities				
Amsterdam	158	9.7	132	10.7
Basel	125	7.7	71	5.8
Berlin	190	11.7	107	8.7
Frankfurt	163	10.0	111	9.0
Linz	146	9.0	84	6.8
Paris	158	9.7	136	11.1
Rotterdam	191	11.7	174	14.2
Strasbourg	184	11.3	168	13.7
Vienna	188	11.6	167	13.6
Zurich	124	7.6	79	6.4
N	1627		1229	

Source: TIES surveys 2007/2008

**Table 9:** Binary logistic regression: Interplay between social identifications, country contexts and local voting probability

	Model A1	Model A2
Social Identification		
Turkish identification	.25 (.25)	.19 (.11)†
Islamic identification	−.01 (.07)	−.05 (.02)*
Perceived Discrimination		
Group discrimination of Turkish-origin individuals	.08 (.09)	.06 (.07)
Group discrimination of Muslims	−.08 (.07)	−.09 (.08)
Personal experiences of discrimination	.02 (.07)	.04 (.06)
Control variables		
Age	.07 (.02)***	.08 (.02)***
Women (ref. men)	.04 (.12)	−.02 (.12)
Middle education (ref. lower)	−.33 (.42)	−.16 (.49)
Higher education (ref. lower)	.24 (.51)	.37 (.65)
Hours worked per week	.00 (.01)	.01 (.01)
Monthly income	−.05 (.05)	−.07 (.06)
Language proficiency	.12 (.03)***	.11 (.04)**
National identification	.26 (.11)*	.21 (.10)*
National-origin composition neighborhood	−.03 (.07)	−.02 (.07)
Turkish best friend (ref. native best friend)	−.57 (.20)**	−.37 (.20)†
Other origin best friend (ref. native best friend)	−.14 (.22)	.16 (.20)
Islamic denomination (ref. Sunni)		
Shia	.72 (.46)	.76 (.45)†
Alevi	.32 (.29)	.43 (.29)
Other Muslims	−.11 (.11)	−.11 (.11)
Non-believer	.16 (.17)	
Countries (ref. Austria)		
France	.22 (1.50)	−.05 (.64)
Germany	.95 (1.36)	−1.18 (.39)**
Switzerland	.65 (1.20)	−.12 (.91)
The Netherlands	.99 (1.21)	−.35 (.43)
Interaction effects		
France * Turkish identification	−.06 (.29)	
Germany * Turkish identification	−.36 (.25)	
Switzerland * Turkish identification	−.17 (.25)	
The Netherlands * Turkish identification	−.03 (.23)	
France * Islamic identification		.05 (.10)
Germany * Islamic identification		.15 (.04)***
Switzerland * Islamic identification		.07 (.13)
The Netherlands * Islamic identification		.31 (.11)**
N	1627	1229
Df	33	31
Prob>Chi2	.000	.000

The results are reported in log odds, the standard errors are indicated in parentheses and the standard errors are clustered on cities

Source: TIES survey 2007/2008; † *p* < 0.10 **p* < 0.05 ***p* < 0.01 ****p* < 0.001

**Table 10:** Binary logistic regression: Interplay between social identifications, group discrimination and local voting probability

	Model A3	Model A4
Social Identification		
Turkish identification	−.12 (.19)	.20 (.11)†
Islamic identification	.02 (.06)	.20 (.13)
Perceived Discrimination		
Group discrimination of Turkish-origin individuals	−.21 (.23)	.06 (.07)
Group discrimination of Muslims	−.08 (.07)	.09 (.16)
Personal experiences of discrimination	.02 (.07)	.04 (.06)
Control variables		
Age	.07 (.02)***	.08 (.02)***
Women (ref. men)	.04 (.12)	−.01 (.12)
Middle education (ref. lower)	−.30 (.40)	−.20 (.48)
Higher education (ref. lower)	.25 (.51)	.31 (.63)
Hours worked per week	.00 (.01)	.01 (.01)
Monthly income	−.04 (.06)	−.08 (.06)
Language proficiency	.13 (.03)***	.11 (.04)**
National identification	.25 (.11)*	.21 (.10)*
National-origin composition neighborhood	−.03 (.07)	−.02 (.07)
Turkish best friend (ref. native best friend)	−.58 (.19)**	−.36 (.21)†
Other origin best friend (ref. native best friend)	−.18 (.21)	.14 (.21)
Islamic denomination (ref. Sunni)		
Shia	.62 (.38)	.72 (.44)
Alevi	.40 (.28)	.45 (.29)
Other Muslims	−.10 (.10)	−.09 (.10)
Non-believer	.22 (.17)	/
Countries (ref. Austria)		
France	−.00 (.33)	.14 (.27)
Germany	−.37 (.41)	−.56 (.36)
Switzerland	−.02 (.49)	.20 (.45)
The Netherlands	.91 (.34)**	.94 (.30)**
Interaction effects		
Turkish identification*Group discrimination of Turkish-origin individuals	.07 (.04)	
Islamic identification*Group discrimination of Muslims		.04 (.03)
N	1627	1229
Df	30	28
Prob>Chi2	.000	.000

The results are reported in log odds, the standard errors are indicated in parentheses and the standard errors are clustered on cities

Source: TIES survey 2007/2008; † *p* < 0.10 **p* < 0.05 ***p* < 0.01 ****p* < 0.001

**Table 11:** Binary logistic regression: Interplay between social identifications, personal discrimination and local voting probability

	Model A5	Model A6
Social Identification		
Turkish identification	−.03 (.21)	.20 (.10)*
Islamic identification	.02 (.06)	.30 (.13)*
Perceived Discrimination		
Group discrimination of Turkish-origin individuals	.07 (.09)	.05 (.07)
Group discrimination of Muslims	−.08 (.08)	−.08 (.08)
Personal experiences of discrimination	−.22 (.32)	.59 (.20)**
Control variables		
Age	.07 (.02)***	.08 (.02)***
Women (ref. men)	.05 (.12)	−.00 (.12)
Middle education (ref. lower)	−.31 (.41)	−.19 (.49)
Higher education (ref. lower)	.23 (.51)	.33 (.63)
Hours worked per week	.00 (.01)	.01 (.01)
Monthly income	−.04 (.06)	−.07 (.06)
Language proficiency	.12 (.03)***	.11 (.04)**
National identification	.25 (.11)*	.21 (.11)*
National-origin composition neighborhood	−.04 (.07)	−.02 (.07)
Turkish best friend (ref. native best friend)	−.57 (.20)**	−.38 (.22)†
Other origin best friend (ref. native best friend)	−.16 (.21)	.13 (.20)
Islamic denomination (ref. Sunni)		
Shia	.59 (.38)	.73 (.44)†
Alevi	.38 (.28)	.46 (.28)
Other Muslims	−.10 (.10)	−.08 (.10)
Non-believer	.23 (.17)	/
Countries (ref. Austria)		
France	.00 (.33)	.17 (.27)
Germany	−.37 (.41)	−.52 (.36)
Switzerland	−.01 (.49)	.23 (.45)
The Netherlands	.91 (.34)**	.97 (.30)**
Interaction effects		
Turkish identification*Personal discrimination	.06 (.07)	
Islamic identification*Personal discrimination		−.13 (.04)**
N	1627	1229
Df	30	28
Prob>Chi2	.000	.000

The results are reported in log odds, the standard errors are indicated in parentheses and the standard errors are clustered on cities

Source: TIES survey 2007/2008; † *p* < 0.10 **p* < 0.05 ***p* < 0.01 ****p* < 0.001

**Table 12:** Binary logistic regression: Interplay between social identifications, group discrimination, personal discrimination and local voting probability

	Model A7	Model A8
Social Identification		
Turkish identification	−.24 (.41)	.21 (.11)*
Islamic identification	.02 (.06)	.01 (.48)
Perceived Discrimination		
Group discrimination of Turkish-origin individuals	−.21 (.44)	.06 (.08)
Group discrimination of Muslims	−.08 (.07)	−.35 (.61)
Personal experiences of discrimination	−.24 (1.03)	−.10 (1.12)
Control variables		
Age	.07 (.02)***	.08 (.02)***
Women (ref. men)	.04 (.12)	−.00 (.12)
Middle education (ref. lower)	−.32 (.41)	−.19 (.52)
Higher education (ref. lower)	.23 (.52)	.32 (.66)
Hours worked per week	.00 (.01)	.01 (.01)
Monthly income	−.05 (.06)	−.07 (.06)
Language proficiency	.13 (.03)***	.12 (.04)**
National identification	.25 (.11)*	.21 (.11)*
National-origin composition neighborhood	−.03 (.07)	−.02 (.07)
Turkish best friend (ref. native best friend)	−.57 (.19)**	−.38 (.21)†
Other origin best friend (ref. native best friend)	−.17 (.21)	.12 (.20)
Islamic denomination (ref. Sunni)		
Shia	.64 (.40)	.75 (.48)
Alevi	.40 (.28)	.46 (.29)
Other Muslims	−.10 (.11)	−.08 (.10)
Non-believer	.22 (.17)	/
Countries (ref. Austria)		
France	−.01 (.32)	.16 (.27)
Germany	−.38 (.41)	−.52 (.36)
Switzerland	−.03 (.48)	.22 (.44)
The Netherlands	.90 (.34)**	.97 (.31)**
Interaction effects		
Turkish identification * Group discrimination of Turkish-origin individuals	.08 (.08)	
Turkish identification * Personal discrimination	.09 (.21)	
Group discrimination of Turkish-origin individuals * Personal discrimination	.03 (.25)	
Turkish identification * Group discrimination of Turkish-origin individuals * Personal discrimination	−.01 (.05)	
Islamic identification * Group discrimination of Muslims		.08 (.13)
Islamic identification * Personal discrimination		.07 (.26)
Group discrimination of Muslims * Personal discrimination		.17 (.27)
Islamic identification * Group discrimination of Muslims * Personal discrimination		−.05 (.06)
N	1627	1229
Df	33	31
Prob>Chi2	.000	.000

The results are reported in log odds, the standard errors are indicated in parentheses and the standard errors are clustered on cities

Source: TIES survey; † *p* < 0.10 **p* < 0.05 ***p* < 0.01 ****p* < 0.001

## Abbreviations

MIPEX: The Migrant Integration Policy Index
TIES: The Integration of the European Second Generation

## Footnotes

1 Retrieved from www.tiesproject.eu

2 Retrieved from http://www.mipex.eu/political-participation

3 Retrieved from http://www.mipex.eu/play/

4 Retrieved from http://www.mipex.eu/play/.

5 The authors refer to fraternalistic deprivation.

6 Retrieved from http://www.tiesproject.eu

7 Only respondents with citizenship are included for Switzerland due to the cantonal and municipal variation in granting foreigners local voting rights.

8 Feeling connected to the city the respondent lived in was also included as robustness. This indicator did not reach significance and did not alter any of the analyses, so was excluded.
